# Comparative genomics approach to evolutionary process connectivity

**DOI:** 10.1111/eva.12978

**Published:** 2020-05-01

**Authors:** Pierre‐Alexandre Gagnaire

**Affiliations:** ^1^ ISEM Univ Montpellier CNRS EPHE IRD Montpellier France

**Keywords:** comparative population genomics, conservation and management, demographic history, genetic connectivity, life history traits, whole‐genome resequencing

## Abstract

The influence of species life history traits and historical demography on contemporary connectivity is still poorly understood. However, these factors partly determine the evolutionary responses of species to anthropogenic landscape alterations. Genetic connectivity and its evolutionary outcomes depend on a variety of spatially dependent evolutionary processes, such as population structure, local adaptation, genetic admixture, and speciation. Over the last years, population genomic studies have been interrogating these processes with increasing resolution, revealing a large diversity of species responses to spatially structured landscapes. In parallel, multispecies meta‐analyses usually based on low‐genome coverage data have provided fundamental insights into the ecological determinants of genetic connectivity, such as the influence of key life history traits on population structure. However, comparative studies still lack a thorough integration of macro‐ and micro‐evolutionary scales to fully realize their potential. Here, I present how a comparative genomics framework may provide a deeper understanding of evolutionary process connectivity. This framework relies on coupling the inference of long‐term demographic and selective history with an assessment of the contemporary consequences of genetic connectivity. Standardizing this approach across several species occupying the same landscape should help understand how spatial environmental heterogeneity has shaped the diversity of historical and contemporary connectivity patterns in different taxa with contrasted life history traits. I will argue that a reasonable amount of genome sequence data can be sufficient to resolve and connect complex macro‐ and micro‐evolutionary histories. Ultimately, implementing this framework in varied taxonomic groups is expected to improve scientific guidelines for conservation and management policies.

## 1 INTRODUCTION

Anthropogenic landscape alterations affect all strata of marine and terrestrial ecosystems (Boivin et al., 2016; Halpern et al., 2008). The negative effects of human activities are visible at multiple scales, from communities to genes, and cause connectivity disruptions in both its structural and functional dimensions. For instance, habitat fragmentation or the breaking of physical links connecting habitats patches can affect landscape connectivity, which refers to measurable physical connectivity between habitat patches in a landscape. But in addition to this, the way in which a given landscape is perceived from the “species‐eye” view can lead to a more complex canvas of habitat connectivity (Lindenmayer & Fischer, 2007). Species‐specific constrained ability to disperse through the landscape may indeed generate additional reductions in connectedness between patches of suitable habitat, due for instance to behavioral components. Therefore, the physical spatial structure of a landscape can impose different connectivity constraints, depending on species.

The conservation of connectivity among patches of suitable habitat is a well‐established measure to limit the potentially detrimental impacts of landscape disruption in conservation biology. However, the extent to which landscape connectivity is essential to combat biodiversity loss remains unclear (Crooks & Sanjayan, 2006). On one hand, corridors have been used with success to facilitate dispersal among patches of fragmented habitats, providing demographic benefits to connected patches (Beier & Noss, 1998) and mitigating the erosion of genetic diversity (Christie & Knowles, 2015). On the other hand, increased connectivity sometimes comes with genetic and demographic costs, such as disruption of local adaptation, increased risks of genetic swamping, detrimental hybridization, introduction of alien species or transmission of contagious diseases (Simberloff & Cox, 1987). Managing connectivity for a given species is therefore a balancing act. Not only because other species occupying the same landscape may experience connectedness in different ways, but also because the eco‐evolutionary consequences of connectedness can strongly differ from one species to another. The evaluation of species‐specific connectivity needs and consequences thus remains a major challenge to be addressed.

Characterizing the different aspects of connectivity for a particular species in a given landscape requires ecological and genetic data. The process‐oriented frameworks of metapopulation ecology and genetics allow quantifying relevant ecological and evolutionary parameters at different spatial scales (Moilanen & Hanski, 2001). The two approaches, however, differ in how they consider and measure connectivity (Lowe & Allendorf, 2010). Metapopulation ecology mainly considers the relative contribution of dispersal to population growth and vital rates to assess demographic connectivity. By contrast, the population genetics approach focuses on gene flow. Genetic connectivity studies typically evaluate the extent to which the contribution of migrants' genes to a recipient gene pool affects population genetic diversity, integrity, and evolutionary potential (Sgro, Lowe, & Hoffmann, 2011). Although complementary, ecological and genetic frameworks do not cover the same time scales. While demographic connectivity is mainly about the contribution of contemporary dispersal to species persistence, the genetic approach captures the effect of evolutionary processes acting at different time periods from the distant past to the present. This difference in timescales has hindered the combination of ecological and genetic approaches in connectivity studies (Cayuela et al., 2018).

Another source of difficulty stems from the fact that, in practice, individuals of a given species cannot be considered equivalent to each other, as it is often assumed in demographic and genetic connectivity models. Just because different populations of the same species usually display different local adaptations, different levels of genetic load, or even genetic incompatibilities, crosses between immigrants and residents are often not neutral and the consequences for the fitness of outbred descendants can be varied. Population genomic approaches now able to capture part of this information directly from the analysis of genome sequences, a task that is greatly facilitated by taking into account the demographic history of populations. However, studies of contemporary connectivity accounting for the long‐term evolutionary history of the species remain scarce. The field of comparative phylogeography has already started to address part of these issues (Bermingham & Moritz, 1998). But further integration is needed to understand what kinds of interactions between biological parameters and historical contingencies shape the current diversity of species' evolutionary responses to a shared landscape.

The objective of this review is to emphasize the need and propose possible directions toward combining macro‐ and micro‐evolutionary scales in genetic connectivity research to facilitate this integration. In order to focus on timescales and processes, the term “evolutionary process connectivity” (Worboys, Francis, & Lockwood, 2010) will be used to refer to spatially dependent evolutionary processes pertaining to both macro‐ and micro‐evolutionary scales. This concept embraces a large diversity of spatially based processes including population structure, local adaptation, genetic admixture, and speciation, which all lie at the core of genetic connectivity research. The following sections will start with a rapid overview of the diversity of evolutionary processes attainable with genetic approaches, and the importance of interrogating past demographic history to understand the contemporary consequences of genetic connectivity. The benefits of a comparative genomics framework will be finally considered to compare species evolutionary responses to spatially structured landscapes and attempt to relate this diversity to species biology and ecology.

## 2 OVERVIEW OF MOLECULAR APPROACHES TO EVOLUTIONARY PROCESS CONNECTIVITY

Spatially dependent evolutionary processes have been intensely studied using molecular markers, although with different degrees of spatial and temporal resolution (Fenderson, Kovach, & Llamas, 2020; Guillot, Leblois, Coulon, & Frantz, 2009; Manel, Schwartz, Luikart, & Taberlet, 2003; Waples & Gaggiotti, 2006). The last decade has been particularly marked by a significant increase in the density of markers used in studies of wild nonmodel species, from about one marker to several thousand per chromosome (Funk, McKay, Hohenlohe, & Allendorf, 2012), and culminating even more recently in the use of complete genomic sequences (Ellegren, 2014). Thus, the range of approaches available in the molecular ecologists' toolbox now allows empirical studies to be adapted to the level of spatial, temporal, and genomic resolution required to study the intended evolutionary process.

### 2.1 Oligo‐marker approaches

**FIGURE 1:**
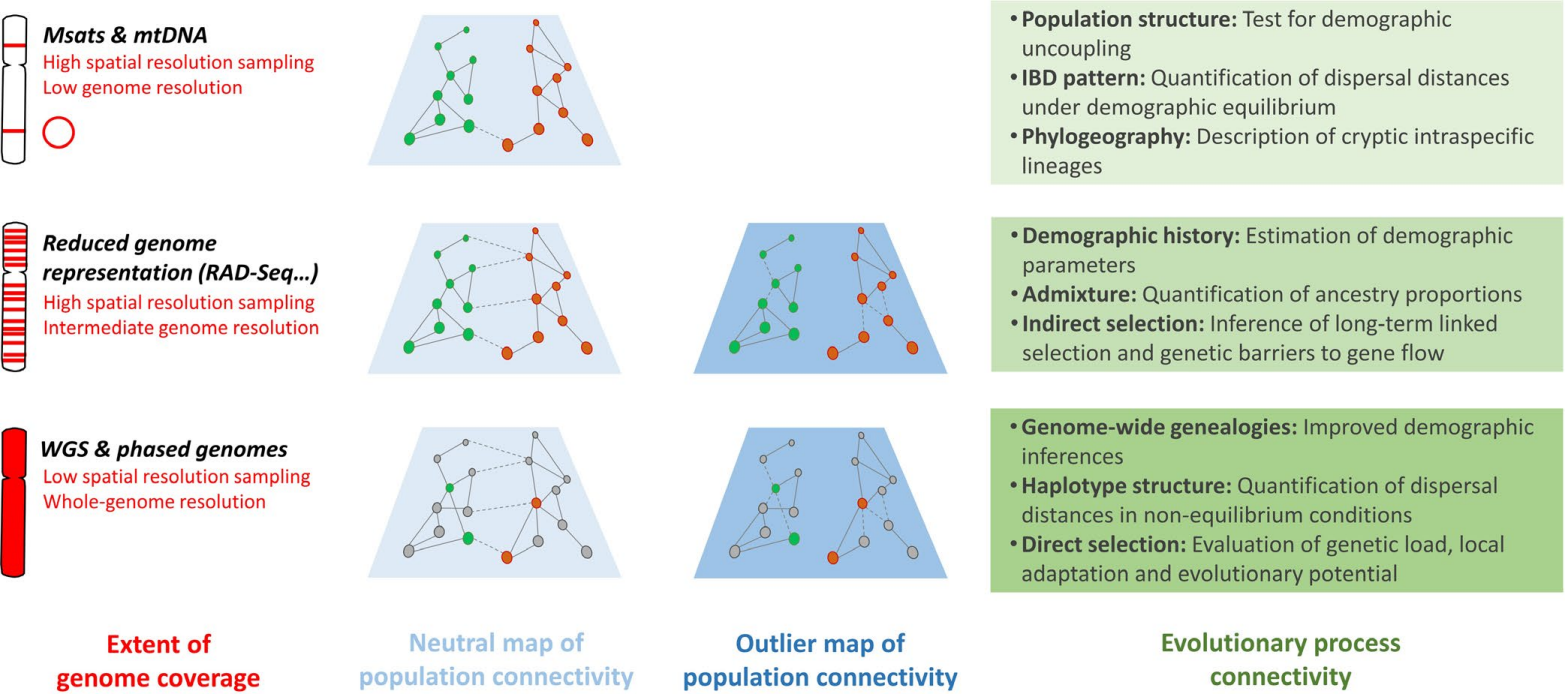
Molecular approaches to evolutionary process connectivity. The necessary compromise between the extent of genome coverage and the degree of spatial resolution sampling constrains the diversity of evolutionary processes attainable with molecular approaches. This review particularly considers the contribution of whole‐genome sequencing approaches to our understanding of evolutionary process connectivity. Colored dots within connectivity maps may refer to population samples or to individuals

Oligo‐marker approaches (i.e., based on about 100 makers or less) potentially provide high‐spatial resolution neutral maps of population genetic connectivity (Figure 1). The use of small marker datasets may be the only strategy compatible with budget limitations when it is necessary to analyze thousands of samples to quantify contemporary dispersal in parentage (Baetscher et al., 2019; Moore, Draheim, Etter, Winterstein, & Scribner, 2014) or genetic assignment studies (Johansson et al., 2018). Provided that a sufficient level of genetic differentiation exists among populations, the description of the fine‐scale genetic structure makes it possible to test for demographic uncoupling. For instance, Nykänen et al. (2019) showed that genetically differentiated populations of bottlenose dolphins (*Tursiops truncatus*) from the Northwestern Atlantic are connected by very low migration rates (<1%), a value too low to assume demographic cohesiveness (Hastings, 1993).

Oligo‐marker approaches are also well suited for inferring long‐term genetic connectivity using indirect methods. For instance, the standard deviation of parent–offspring dispersal distances (*σ*) can be estimated from isolation‐by‐distance patterns (Rousset, 1997), and the absolute number of migrants per generation (*N*_e_ *m*) can be inferred from genetic differentiation measures such as *F*_ST_ under some assumptions (reviewed in Broquet & Petit, 2009). At an even deeper temporal scale, a few gene sequences can be sufficient to detect molecular divergence. Phylogeographic studies that typically rely on mitochondrial data have revealed the pervasive effects of quaternary climate oscillations on lineage diversification in many taxa (Avise, 2000; Bernatchez & Wilson, 1998; Hewitt, 2004) (Box 1). For instance, a recent meta‐analysis of more than 15,500 COI sequences recently showed that European butterflies have massively undergone glacial isolations followed by postglacial expansions (Dapporto et al., 2019). Therefore, the neutral maps of evolutionary process connectivity that can be obtained with a handful of loci already cover a wide range of micro‐ and macro‐evolutionary processes (Figure 1).

Box 1 **Bridging comparative phylogeography, population and conservation genomics: an important chapter in Louis Bernatchez's school**

I met Louis in 2007 at the European Ichthyological Society congress in Dubrovnik. I was a PhD student working on speciation in tropical eels and attending my first congress. Louis had just published an important study on hybridization between American and European eels using AFLPs (Albert, Jónsson, & Bernatchez, 2006). At the time, AFLPs were markers of choice for identifying selection footprints in the genomes of nonmodel species (Campbell & Bernatchez, 2004; Wilding, Butlin, & Grahame, 2001). I introduced myself to Louis at the congress social evening to ask him if he had looked at how introgression rates vary among AFLP loci between the two Atlantic eel species. He replied very simply “No, but you could come to my lab and do it!”. The deal was made just like that, and four months later I was in Quebec to do those analyses. Later, I returned to his laboratory as a post‐doc and spent more than 2 years of intense scientific life there. It was a humanly enriching experience that remains today a true source of inspiration in my scientific career. For all this, and for all the good times shared since then, I thank you and wish you a happy birthday Louis!

One of the many research projects in progress when I was in Louis' laboratory was a comparative genetic study of North American freshwater fishes (April, Hanner, Mayden, & Bernatchez, 2013; April, Mayden, Hanner, & Bernatchez, 2011). It followed a seminal comparative study of mitochondrial phylogeography which, more than a decade earlier, had shown the impact of Pleistocene glacial cycles on lineage diversification in Nearctic and Palearctic fishes (Bernatchez & Wilson, 1998). The running project aimed to push a step further by combining mitochondrial and AFLP data in several species in parallel, in order to better document genetic exchanges in the contacts zones between Mississippian and Atlantic glacial lineages. The results showed that most of the divergent glacial lineages have remained partially reproductively isolated despite hybridization (April, Hanner, Dion‐Côté, & Bernatchez, 2013). This study reinforced the idea that alternating episodes of isolation and contact caused by glacial cycles have initiated and fostered speciation in multiple fish species in North America.

Another important contribution of this research was to reveal the existence of morphologically cryptic evolutionary lineages, which are relevant for conservation strategies in a context of large‐scale species introductions and extirpations. The development of evolutionary applications in conservation biology has long been encouraged by Louis' scientific initiatives and research program. The purpose of this review is to encourage continued efforts to link comparative phylogeography and population genomics to address conservation issues related to connectivity.

### 2.2 Multi‐marker approaches

The most important contribution of the genomic revolution to the study of these processes is the several orders of magnitude increase in the quantity and density of genetic polymorphism data (i.e., several thousands to several millions; Figure 1). This increase has had two advantageous consequences. The first was to access many independent markers across the genome, each of which carries some of the information describing the coalescence process (Rosenberg & Nordborg, 2002). Because there are such a high number of possible random gene genealogies for a given sample with a given demographic history, only a deep sampling of genomic variation can accurately capture the stochasticity of the coalescence process. Population genomics approaches have thus benefited to both recent and historical demographic inferences, providing improved maps of neutral evolutionary processes connectivity.

At a small temporal scale, they provide a bridge between parentage methods and analyses of isolation‐by‐distance patterns by extending the range of pedigrees for which reliable genetic relatedness values can be obtained. For instance, Aguillon et al. (2017) used a pattern of decreased genetic relatedness with increased geographic distance to infer recent demography in the Florida scrub‐jay (*Aphelocoma coerulescens*).

At longer time scales, historical demography can be learnt from genome‐wide polymorphism data using diverse inference frameworks such as full‐likelihood (Hey & Nielsen, 2007), composite‐likelihood (Excoffier, Dupanloup, Huerta‐Sánchez, Sousa, & Foll, 2013; Gutenkunst, Hernandez, Williamson, & Bustamante, 2009), or approximate Bayesian computation (Beaumont, Zhang, & Balding, 2002). The strength of these approaches is that they make it possible to dissociate the effect of drift captured by the effective population size parameter (*N*_e_) and the per‐generation migration rate (*m*) on gene flow (*N*_e_ *m*). Thus, the effect of time on allele frequency changes can be represented on a genetic drift intensity scale, and evolutionary independence can be assessed from estimated migration rates (Hey & Pinho, 2012). To handle the large amount of information in large population genomic datasets, demographic inference methods generally use summary statistics of the data. A powerful summary statistics that captures many aspects of a species historical demography is the site frequency spectrum (SFS), which conveniently summarizes allele frequency data obtained from reduced representation genome sequencing data (such as RAD‐Seq, ddRAD‐Seq, or GBS) in one or multiple populations. With this type of approach, the spatial sampling resolution required for inferring long‐term migration rates is much coarser (i.e., population samples taken from a few representative locations) than for analyzing contemporary dispersal from geographic patterns of relatedness. Moreover, demographic inferences enable testing for gene flow between demographic entities that rarely exchange genes in nature, such as populations or evolutionary lineages separated by a physical barrier to dispersal or a tension zone. In these situations, migrant individuals or their hybrid offspring cannot be observed directly, but their effective genetic contribution to a recipient population can still be assessed indirectly. This has important implications for understanding the evolutionary consequences of connectedness. For instance, relatively small effective migration rates are sufficient for the spread of slightly advantageous alleles through a physical or a genetic barrier (Barton & Bengtsson, 1986; Piálek & Barton, 1997).

The second most important advantage of increasing marker density has been to facilitate the detection of selective effects through linkage disequilibrium between selected sites and marker loci. Here again, different processes acting at different time scales can be studied for assessing connectivity (Gagnaire et al., 2015). Population genomic approaches to contemporary local adaptation have become increasingly popular thanks to the development of next generation sequencing technologies (Savolainen, Lascoux, & Merilä, 2013; Stapley et al., 2010) and a vast panel of accompanying statistical methods (e.g., Foll & Gaggiotti, 2008; Gautier, 2015; Luu, Bazin, & Blum, 2017). The power of these “genome scan” approaches to document species adaptations and evolutionary potential is constrained by sampling design, population structure, demographic history, and the source and effect size of adaptive mutations (Gagnaire & Gaggiotti, 2016; Lotterhos & Whitlock, 2014, 2015; Wellenreuther & Hansson, 2016). They nevertheless provide efficient means to understand the environmental correlates of population structure and to map the genomic bases of adaptation in different ecological contexts (e.g., Benestan et al., 2016; Hancock et al., 2011; Oziolor et al., 2019; Schweizer et al., 2019).

A notable difficulty in interpreting the results of genome scans for local adaptation is to identify the nature of the evolutionary processes underlying the detection of candidate loci (Bierne, Roze, & Welch, 2013). While abnormally strong spatial structure or association with environmental variables may indicate contemporary local adaptation at outlier markers or closely linked loci, it may also reveal the signature of more ancient processes. For example, the coupling between local adaptation loci and reproductive isolation loci involved in intrinsic pre‐ or postzygotic barriers can generate pervasive genotype‐environment associations across the genome (Bierne, Welch, Loire, Bonhomme, & David, 2011). This type of situation, frequently found between partially reproductively isolated evolutionary entities (e.g., geographical lineages, ecotypes, host races, or cryptic species), is often characterized by a large fraction of the genome associated with signatures of local selection in genome scan studies. Even if contemporary ecological processes (e.g., causing low population density area, or ecotone between ecologically different habitats) can explain the spatial or ecological structure observed at outlier loci, older evolutionary processes often explain their pervasiveness across the genome as well as their origin. This has been shown for instance in some cases of parallel phenotypic divergence among replicate ecotype pairs, whereby anciently diverged geographic lineages have secondarily re‐admixed before a recent spatial reassortment of the same divergently evolved alleles through repeated selection (Le Moan, Gagnaire, & Bonhomme, 2016; Rougemont et al., 2017; Rougeux, Bernatchez, & Gagnaire, 2017; Van Belleghem et al., 2018). Such complex evolutionary scenarios that mix evolutionary processes acting at different time scales make a strong case for the need to infer the demographic history of populations in a more systematic and thorough way.

## 3 SETTING THE SCENE BY MODELING THE EVOLUTIONARY HISTORY

Disentangling demographic from selective effects is an important prerequisite for (a) identifying selected loci in population genomic data, but also for (b) the fundamental interest of understanding the demographic history of populations per se (Excoffier et al., 2013). Teasing apart these effects remains a challenging issue, since the rationale of historical demographic inference is that genome‐wide marker information reflects demography while being robust to selective effects. Some approaches have been proposed to remove the most extremely differentiated loci before building a null model from which the loci involved in local adaption could be more reliably detected (e.g., Whitlock & Lotterhos, 2015). On the other hand, accumulating evidence from empirical population genomic studies indicate that even the core of the distribution of observed statistics such as heterozygosity or differentiation indices can be influenced by selection. For instance, the positive correlation detected between local recombination rate and genetic diversity across the genome of many species indicates that few regions of the genome are completely free from the effects of linked selection (Corbett‐Detig, Hartl, & Sackton, 2015; Sella, Petrov, Przeworski, & Andolfatto, 2009). Therefore, a significant fraction of the genome can potentially depart from the average neutral demographic history. This has been seen as a strong limitation for the ability of molecular polymorphism data to reveal the true demographic history of populations, because selection and demography can leave similar footprints in patterns of genetic variation (Hahn, 2008; Schrider, Shanku, & Kern, 2016). A joint estimation of the demographic and selective effects is therefore highly desirable (Li et al., 2012; Stephan, 2015, 2019).

Different types of selective effects need to be accounted for to achieve this goal (Cruickshank & Hahn, 2014; Cutter & Payseur, 2013; Stephan, 2019). First, selective sweeps causing genetic diversity reductions near selected loci that recently fixed beneficial mutations (Maynard Smith & Haigh, 1974). When recurrent selective sweeps occur, genomic regions of reduced recombination rates are expected to display reduced levels of diversity (Kaplan, Hudson, & Langley, 1989). However, this may be confounded by the effect of background selection against recurrent deleterious mutations, which also reduces the level of variation at linked neutral sites on a scale that depends on the rate of recombination and gene density (Charlesworth, Morgan, & Charlesworth, 1993; Hudson & Kaplan, 1995; Nordborg, Charlesworth, & Charlesworth, 1996). Estimating the joint effects of selective sweeps and background selection on neutral variation has been the focus of several theoretical and empirical works (Comeron, 2014; Elyashiv et al., 2016; Kim & Stephan, 2000; Lohmueller et al., 2011). Among them, studies in *Drosophila* have highlighted the importance of considering linked selection, in particular by including the unavoidable effects of background selection, for the purpose of making demographic inferences and genome scans of selection (Comeron, 2017).

Applying the sophisticated approaches developed for the study of model species such as flies can be out of reach in nonmodel organisms that still lack functionally annotated genomes and genetic maps. Moreover, an explicit treatment of linked selection within complex historical demographic models accounting for phases of divergence and admixture remains highly challenging. A possible alternative to account for linked selection in demographic inference is to capture its genomically localized indirect effect on the reduction of genetic diversity. This effect is usually described as being equivalent to a local reduction in *N*_e_, at least as it concerns the effect of background selection (Burri, 2017; Charlesworth, 2009). Different approaches modeling among‐locus variation in genetic drift have been developed and applied to various organisms, sometimes assuming complex historical demographic models (Rougeux et al., 2017; Roux et al., 2016; Sousa, Carneiro, Ferrand, & Hey, 2013). They revealed that genome‐wide variation in *N*_e_ due linked selection shapes genome diversity patterns over different time frames, from within‐population to between‐species levels. This is also reflected by the finding of correlated genomic landscapes of genetic diversity summary statistics in birds, across different phylogenetic scales ranging from populations of the same species to distantly related species (Vijay et al., 2017). Existing modeling frameworks thus enable us to infer null models of demographic history that account for linked selection, even in nonmodel species.

Relaxing the assumption that all loci share the same demography has also allowed to capture the effect of genetic barriers to gene flow between populations in demographic divergence models (Roux, Tsagkogeorga, Bierne, & Galtier, 2013; Sousa et al., 2013; Tine et al., 2014). Selection against foreign alleles causing maladaptation in hybrids or migrant genotypes generates local reduction in effective migration rate (*m*_e_) at linked neutral markers, on a chromosomal scale that depends on recombination (Barton & Bengtsson, 1986). Accounting for heterogeneous *m*_e_ in addition to *N*_e_ across the genome in population genomic studies has helped dissociating the effects of linked selection and genetic barriers to gene flow on divergence. For instance, the analysis of 61 pairs of populations/species of animals within this framework revealed that the probability of gene flow between diverging entities is significantly reduced above 2% of net molecular divergence (Roux et al., 2016). The range of net divergence values from 0.5% to 2%, where many cryptic species lie, is often characterized by heterogeneous gene flow across semi‐permeable genomes. This has important implications for the purpose of this review, since genetic subdivisions located in this intermediate “gray zone” of speciation may have been initiated by factors that differ from those affecting contemporary connectivity patterns. Historical demographic inference is therefore a key approach for the integration of macro‐ and micro‐evolutionary scales in the study of genetic connectivity.

## 4 ASSESSING THE EVOLUTIONARY CONSEQUENCES OF CONTEMPORARY GENETIC CONNECTIVITY

Understanding the importance of connectivity for biodiversity conservation requires assessing the extent to which increased connectivity is favorable, or on the contrary unfavorable, to the objectives set in conservation biology. I will not reconsider here the question of the demographic impact of connectivity on population persistence. Instead, I will focus on the evolutionary consequences that depend directly on gene flow following effective migration among populations within a landscape. As we saw before, this may involve complex interactions between demographic and selective forces throughout the history of populations.

Several important aspects pertaining to the evolutionary consequences of contemporary genetic connectivity have already been considered extensively in the literature of genetic rescue (Bell et al., 2019; Tallmon, Luikart, & Waples, 2004; Whiteley, Fitzpatrick, Funk, & Tallmon, 2015) and assisted gene flow (Aitken & Whitlock, 2013). Here, I consider how different types of interactions between selected mutations and recombination determine the outcome of gene flow, as genetic fragments of different ancestries mix and progressively recombine across generations.

**FIGURE 2:**
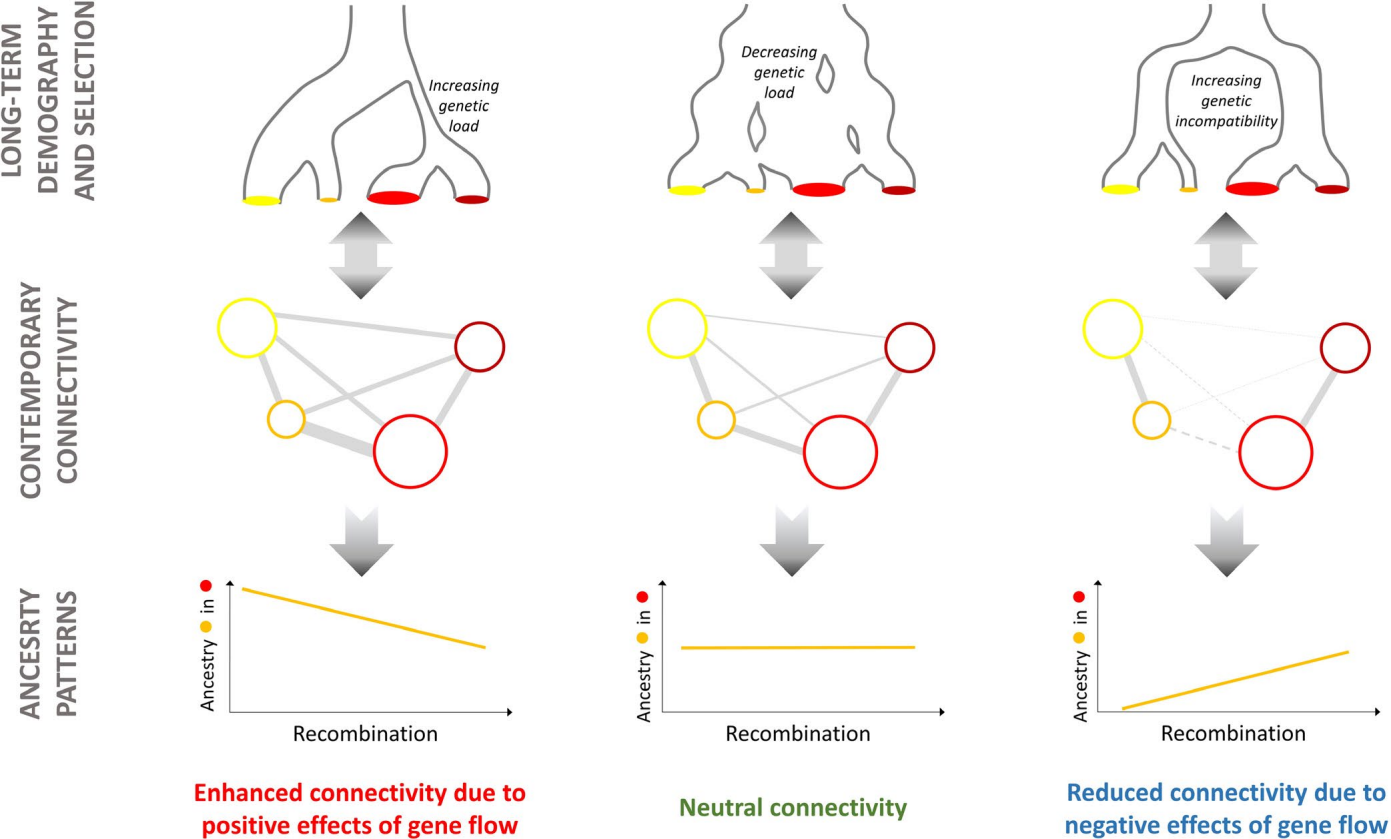
Integration of micro‐ and macro‐evolutionary time scales. During the long‐term evolutionary history of populations, ancestral genetic variation is sorted at different rates among descendant populations, possibly accelerated by linked selection or braked by gene flow. Depending on demographic conditions, the degree of local adaptation to the local environment, the amount of genetic load, or reproductive isolation barriers with other such populations may differ among populations. These processes impact the evolutionary outcomes of contemporary connectivity, resulting in more or less discernable footprints in genome polymorphism data. Here, the outcome of different imaginary demographic histories on genome‐wide correlation between introgressed ancestry and recombination are illustrated, focusing on contemporary gene flow from the orange into the red population. Left: Genetic load has increased in one lineage after a period of bottleneck followed by recent expansion. Middle: A growing metapopulation has purged its genetic load explained by recessive mutations. Right: Two diverging lineages have accumulated genetic incompatibilities during geographical isolation

Perhaps the most frequently observed initial effect of outcrossing between individuals from genetically distinct source populations is heterosis, that is, increased fitness of offspring produced between locals and immigrants compared to their parents. The main mechanism behind heterosis is the masking of partially recessive deleterious mutations due to increased genome‐wide heterozygosity in hybrid offspring (Tallmon et al., 2004). Using forward‐in‐time simulations, Kim, Huber, and Lohmueller (2018) showed that heterosis can lead to a rapid increase in the frequency of introgressed ancestry in the generations directly following admixture. This effect is even stronger in low‐recombining regions of the genome, where the efficacy of purifying selection is lower. Although the effect of heterosis rapidly dissipates in the early generations, the local effect of associative overdominance (i.e., the effect of heterosis at the local scale) will remain for some time relatively stronger within regions of low versus high recombination rates. This leads to a negative correlation between local recombination rate and introgression in the presence of recessive deleterious mutations (*h* = 0), irrespective to the relative amounts of genetic load in the donor and recipient populations (Figure 2).

The outcome of gene flow may differ in the presence of only additive deleterious mutations (*h* = 0.5), that is, in the absence of associative overdominance. In this case, a positive correlation between introgression and recombination can be obtained if the donor population has a lower longer‐term *N*_e_ (i.e., higher genetic load) than the recipient population (Kim et al., 2018; Schumer et al., 2018). In this hybridization load model, hybrids formed in the recipient population suffer from increased genetic load compared to parental genotypes (Schumer et al., 2018), although the relative fitness of the recipient compared to the donor population can be little affected over the long term (Kim et al., 2018). This is because most deleterious introgressed DNA fragments are rapidly purged by selection (i.e., <10 generations) following admixture (Harris & Nielsen, 2016; Veller, Edelman, Muralidhar, & Nowak, 2019). This reasoning considering unconditionally deleterious alleles in the recipient genome can be extended to mutations conferring a local disadvantage to recipient individuals in their local environment. Therefore, qualitatively similar conclusions can be obtained with additive mutations involved in local adaptation (Schumer et al., 2018; Veller et al., 2019).

Alternatively, immigration can also decrease the fitness of hybrid offspring due to negative epistatic interactions with other alleles at other genes. If donor and recipient populations have sufficiently diverged to have evolved genetic incompatibilities, the disruption of co‐adapted gene complexes can result in outbreeding depression (Maheshwari & Barbash, 2011). Simulations under a pairwise genetic incompatibility model showed that selection against recombinant genotypes tend to result in reduced introgressed ancestry within regions of lower cross‐over rates (Schumer et al., 2018). This can be explained by more efficient selection against blocks containing several tightly linked incompatibility alleles of similar ancestries (Barton & Bengtsson, 1986).

The different modes (recessive, additive, epistatic) and mechanisms of action (genetic load, local adaption, incompatibilities) of mutations involved in fitness may be undistinguishable based on the sign of the correlation between recombination and introgressed ancestry alone. However, the recent findings of such correlations in several systems tend to support the polygenic nature of the underlying mechanisms (Duranton et al., 2018; Edelman et al., 2019; Leitwein et al., 2019; Martin, Davey, Salazar, & Jiggins, 2019; Schumer et al., 2018) and the importance of selected mutations density in shaping the outcome of gene flow (Aeschbacher, Selby, Willis, & Coop, 2017). In the future, complementary approaches based on Fisher's geometric model may help disentangle the relative contributions of the different modes and mechanisms of action of mutations in empirical population genomic datasets (Simon, Bierne, & Welch, 2018).

The integration of demographic history and contemporary gene flow studies can help to bridge the gap between macro‐ and micro‐evolutionary scales. This is important to understand the contemporary consequences of genetic connectivity and, in the long term, to predict what might happen in the future. Promising approaches that combine the best of both worlds are being developed (Bradburd, Coop, & Ralph, 2018; Bradburd & Ralph, 2019; Harris, 2019). Among them, methods leveraging tree‐based information from inferred ancestral recombination graphs (Griffiths & Marjoram, 1996) are revolutionizing the analysis of large‐scale genetic variation datasets (Kelleher et al., 2019; Speidel, Forest, Shi, & Myers, 2019). These methods use the complete genealogical information available for each segment of DNA (i.e., between two historical recombination events) genome‐wide to estimate the time to the most recent common ancestor for each pair of individuals at each locus. In doing this, they are able to capture evolutionary processes acting at different time frames, from modern to ancient (although the level of resolution may depend on the amount of individuals). Therefore, temporal dynamics in population sizes and migration rates during demographic history, as well as archaic introgression, can be simultaneously inferred with signatures of natural selection. Until recently, estimating ancestral recombination graphs from a set of DNA sequences was posing prohibitive computational and data storage problems. These issues are now being fixed, opening the door to powerful approaches to study the connectivity of spatially dependent evolutionary processes, even in nonmodel organisms.

## 5 IMPLEMENTING A STANDARDIZED COMPARISON ACROSS MULTIPLE SPECIES

Given the wide diversity and complexity of evolutionary processes potentially involved when migrants effectively transmit their genes, understanding genetic connectivity in nonmodel species may seem a daunting task. Despite all the progress made possible by evolutionary genomics approaches, difficulties persist in generalizing and extrapolating the results of single‐species studies. In particular, it remains difficult to understand how different species perceive the environment they share in common. This important question directly concerns the impact of connectivity conservation strategies on biodiversity. Comparisons across multiple species are thus needed to shed light on this issue.

Several comparative studies and meta‐analyses have been carried out, but few of them combined all the key elements required to address the diversity and the determinants of connectivity patterns among species sharing the same environment. Comparative genomics studies generally aggregate species from diverse geographical and ecological contexts (Delmore et al., 2018; Roux et al., 2016). Works performed on species radiation, such as in *Heliconius* butterfly, Darwin's finches, lake whitefish, or monkey flowers (Kronforst et al., 2013; Lamichhaney et al., 2015; Rougeux et al., 2017; Stankowski et al., 2019), do not cover sufficiently large phylogenetic scales to capture the diversity of species response to shared landscapes. Comparative phylogeographic studies, on the other hand, have successfully documented the existence of concordant phylogeographic patterns matching biogeographic boundaries across a wide range of taxa (e.g., Bowen et al., 2016; Patarnello, Volckaert, & Castilho, 2007). However, despite their fundamental contribution to understanding the impact of historical abiotic factors to the structuring of genetic diversity, they cannot reach the level of genomic resolution required to resolve complex demographic histories. Some phylogeographic studies have taken a step forward by implementing spatially explicit comparative frameworks that revealed important ecological determinants of intraspecific lineage diversification, such as differences in dispersal propensity among species (Burney & Brumfield, 2009; Dapporto et al., 2019; Moritz et al., 2009). As such, they paved the way toward a trait‐based comparative phylogeography, addressing the influence of ecological and life history traits on evolutionary process connectivity (Papadopoulou & Knowles, 2016).

Some pioneering studies have recently started to implement this shift using genome‐wide comparative phylogeographic approaches. They tested for alternative biogeographic hypotheses of diversification (Rincon‐Sandoval, Betancur‐R, & Maldonado‐Ocampo, 2019), multiple cycles of isolation‐contact (He et al., 2018), uncoupling between contemporary connectivity and historical gene flow (Myers et al., 2019; Peñalba, Joseph, & Moritz, 2019), or association between cryptic intraspecific diversity and environmental gradients (Le Moan et al., 2019; Stanley et al., 2018). Two of these studies have even accounted for heterogeneous demographic parameters across the genome in order to capture the effect of semi‐permeability and long‐term linked selection (Le Moan et al., 2019; Peñalba et al., 2019). This has been considered as a key component for establishing a powerful comparative population genomics framework (Burri, 2017).

**FIGURE 3:**
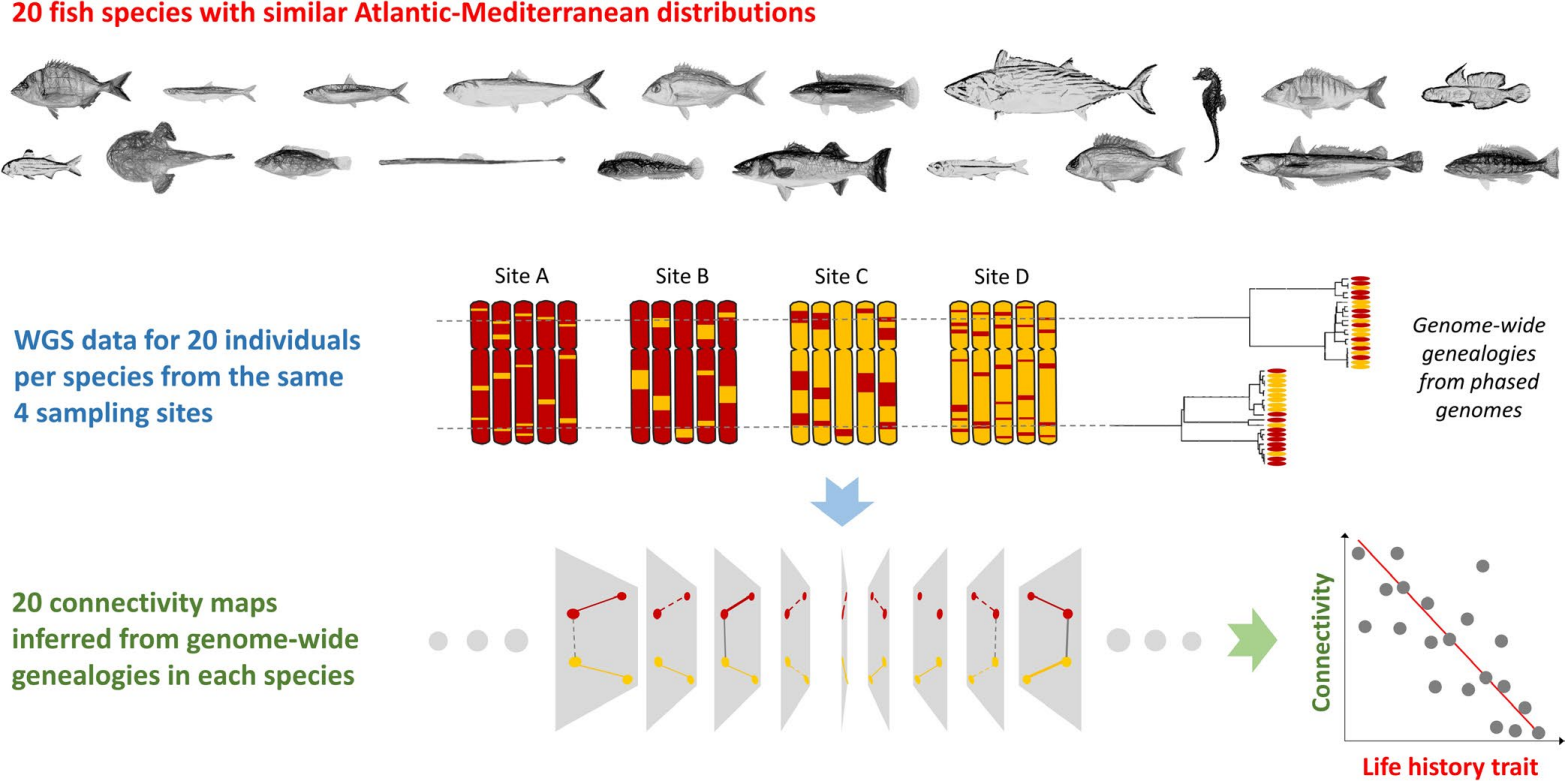
Overview of an ongoing comparative genomic project in Atlantic‐Mediterranean marine fishes with contrasted life history traits. Studied species from top left to lower right: *Diplodus puntazzo*, *Engraulis encrasicolus*, *Sardina pilchardus*, *Alosa fallax*, *Pagellus erythrinus*, *Coris julis*, *Sarda sarda*, *Hippocampus guttulatus*, *Lithognathus mormyrus*, *Gobius niger*, *Mullus surmuletus*, *Lophius budegassa*, *Symphodus cinereus*, *Syngnathus typhle*, *Coryphoblennius galerita*, *Dicentrarchus labrax*, *Atherina boyeri*, *Spondyliosoma cantharus*, *Merluccius merluccius*, *Serranus cabrilla*

The field is now ready for a larger‐scale approach that takes advantage of the latest developments in evolutionary genomics. The proposed strategy is to generate individual whole‐genome sequence data from a limited number of well‐chosen representative locations in each species (Figure 1). The number of sequenced genomes can be significantly scaled down compared to reduced genome representation based studies, typically to a few individuals per location. The rationale behind this is that the loss of precision on allele frequencies estimation will be compensated by access to genome‐wide gene genealogies from phased genomes. The use of haplotype information is expected to improve inferences of long‐term demography and selection, as well as contemporary processes affecting individual fitness in interaction with local recombination rate (Leitwein, Rougemont, Duranton, Gagnaire, & Bernatchez, 2020). Box 2 and Figure 3 sketch out some of the key aspects of a comparative genomics project of this type, recently started in 20 Atlantic‐Mediterranean fish species with contrasted ecological traits.

Box 2 **Comparative genomics approach to evolutionary process connectivity in Atlantic/Mediterranean marine fishes**

Northeastern Atlantic and Mediterranean marine biota share a number of species in common due to multiple events of colonization–extinction–recolonization between basins throughout the complex biogeographical history of this region. The transition zone between the two seas is known as a major phylogeographic break in many temperate Atlantic‐Mediterranean species (Patarnello et al., 2007). This heritage of connectivity variations imposed by paleoclimate fluctuations has led to a wide diversity of contemporary connectivity patterns among marine species, from complete genetic homogeneity to reproductive isolation between closely related species pairs. Comparative studies based on limited numbers of markers have found mixed evidence for the role of life history traits in explaining among‐species differences in connectivity (Dalongeville, Andrello, Mouillot, Albouy, & Manel, 2016; Pascual, Rives, Schunter, & Macpherson, 2017; Patarnello et al., 2007). This may be due to a lack of power to disentangle the confounding effects of multiple evolutionary processes acting at different time scales. Deciphering the relative contribution of historical versus biological and ecological factors could benefit from a switch toward a genome‐scale approach.

The molecular strategy proposed in this review has already been implemented in the European sea bass, *Dicentrarchus labrax*. This species is genetically subdivided into an Atlantic and a Mediterranean lineage, which initially diverged in allopatry around 300,000 BP before undergoing a postglacial secondary contact (Tine et al., 2014). The use of haplotype‐resolved whole‐genome sequences of four to six individuals from three populations (Atlantic, western and eastern Mediterranean) allowed us to reconstruct the demographic divergence history of sea bass lineages, accounting for the effect of linked selection and genetic barriers (Duranton et al., 2018). Low‐recombining regions of the sea bass genome were found to have differentiated faster during geographical isolation due to linked selection, but to have more strongly resisted to gene flow since secondary contact. This finding indicates that partial reproductive isolation has evolved between the two lineages. Analysis of the length distribution of local ancestry tracts revealed a more pronounced introgression from the Atlantic into the Mediterranean than in the opposite direction. The progressive erosion on Atlantic tracts as they diffuse from western to eastern Mediterranean was then used to quantitatively estimate dispersal on an ecologically relevant timescale, using the recombination clock. The spatial scale of dispersal was estimated to <50 km per generation (Duranton, Bonhomme, & Gagnaire, 2019).

Building on these developments in sea bass, we recently launched a comparative study of micro‐ and macro‐evolutionary connectivity patterns in 20 fish species with similar Atlantic‐Mediterranean distributions, but contrasted biological and ecological traits (Figure 3). Our objective is to evaluate the contribution of species life history traits to different evolutionary processes involved in connectivity, both at the within‐population (i.e., within the Atlantic and Mediterranean) and among‐lineages scales. To this end, we began by generating a reference genome assembly for 17 of the 20 species that are currently lacking this important resource. Then, we generated whole‐genome sequence data for 20 individuals per species, evenly taken from the four same sampling sites for all species. In order to optimize the informativeness of gene flow at different spatial scales, we selected one remote site and one site close to the Atlantico‐Mediterranean transition zone in each sea. Using this highly standardized design, we wish to infer the evolutionary history and the contemporary consequences of connectivity from genome‐wide genealogy data for each species. This approach is expected to reveal cryptic species subdivisions that were not previously described. Ultimately, this research should lead to a better understanding of the multiple dimensions of connectivity issues in marine fishes, which could be relevant to fisheries management and biodiversity conservation.

Comparative genomic studies of connectivity need to control as much as possible for potentially confounding factors. For instance, focusing on a single biogeographic context is a necessary condition to reduce the effect of historical contingency, although this cannot be totally eliminated. Other necessary precautions to standardize study design include (but are not necessary limited to) choosing similar sampling locations for all species to compare the effect of homogeneous geographic distances across taxa, and using the same molecular strategy with the same sample size for all sites and species. The repeatability and traceability of bioinformatics pipelines and statistical approaches are also crucial, given the need to execute in parallel numerous steps to move from raw sequence reads to evolutionary parameter inference in each species. The use of workflow management tools (e.g., Snakemake, Nextflow) and containers (e.g., Docker, Singularity) is a good way to achieve exchangeability of analysis software within a collaborative project. The phylogenetic scale is also a matter of concerns for standardization, since overly distant taxa may pose technical difficulties due to strong differences in their genomic architectures (e.g., genome size, repeat content, chromosome number, and recombination landscape).

Finally, different species with contrasted life history traits are likely to display a wide diversity of genetic connectivity patterns. These need to be classified to communicate the results more efficiently and more clearly to managers. The use of reference study systems has been proposed to make progress toward standardized decision‐making for a closely related issue, the delineation of species using genomic data (Galtier, 2019). The idea is that complex evolutionary processes such as speciation, which unfold gradually over time, are not easily compatible with the classification system into discrete entities required to inform conservation policies. Similarly, the use of reference species illustrating important stages within a continuum of well‐documented cases could help describe the diversity of historical and contemporary connectivity patterns across different taxa.

## 6 UNDERSTANDING THE INFLUENCE OF LIFE HISTORY TRAITS ON EVOLUTIONARY PROCESS CONNECTIVITY

How different species with contrasted ecological traits experience connectedness within a similar landscape? To what extent and why do they differ? Linking micro‐ and macro‐evolutionary scales is probably the crux to understanding the diversity of genetic divergence and connectivity patterns and in particular its relationships to species biology and ecology (Harvey, Singhal, & Rabosky, 2019). A comparative framework can provide a robust way to do this (Figure 3, Box 2).

This requires disentangling the influence of species traits on fundamental population genetics parameters that control the efficiency and the pace of population‐scaled demographic and selective processes. For instance, the intensity of genetic drift determines both the level of genetic diversity and the efficiency of selection within populations (Charlesworth, 2009). Comparative genomic studies in plant and animal species have showed that neutral genetic diversity and the amount of weakly deleterious segregating mutations are mostly determined by traits related to parental investment, such as propagule size, fecundity, and longevity (Chen, Glémin, & Lascoux, 2017; Romiguier et al., 2014). The influence of body size, a common proxy for abundance in ecology (White, Ernest, Kerkhoff, & Enquist, 2007), has also been reported at lower phylogenetic scales in European butterflies and Darwin's finches (Brüniche‐Olsen, Kellner, & DeWoody, 2019; Mackintosh et al., 2019). Two species may thus differ in their amount of neutral and weakly deleterious genetic variation due to differences in their life history traits.

Should genetic subdivision occur within such species, the dynamics of divergence would be also impacted by biological and ecological factors influencing genetic drift. Indeed, the rate at which the sorting of ancestral variation occurs within daughter populations is inversely proportional to their effective population sizes. The outcome of short periods of geographic isolation (i.e., less than 10*N*_e_ generations) between two daughter populations in terms of molecular divergence thus depends on the amount of ancestral polymorphism and the rate of lineage sorting during divergence (Arbogast, Edwards, Wakeley, Beerli, & Slowinski, 2002; Edwards & Beerli, 2000), both being under the influence of life history traits. As we saw before, genetic diversity is also determined by the rate at which ancestral variation is erased by linked selection locally in the genome. Since the efficacy of selection against weakly deleterious mutations increases with *N*_e_, linked selection eliminates comparatively more genetic diversity in large compared to small populations (Corbett‐Detig et al., 2015). Therefore, genome‐wide differentiation landscapes could be more strongly impacted by linked selection in abundant compared to rare species. The biological and ecological determinants of ancestral diversity, lineage sorting, and its acceleration through linked selection, probably impact real genomic data through different pathways, themselves influencing *N*_e_ at different time scales. Therefore, understanding the proximal causes of the correlations between life history traits influencing drift and inferred connectivity processes remains a challenging exercise.

As opposed to genetic drift, the homogenizing force of migration reduces the rate at which allele frequencies change between populations undergoing divergence. However, the predicted consequences of dispersal on connectivity patterns are not straightforward either. On the one hand, species traits favoring increased dispersal capabilities (e.g., prolonged larval phase in marine organisms, seed‐dispersal structures in plants) could be associated to increased chances of colonizing isolated habitat patches, maintaining diversified metapopulations in fragmented landscapes (Cahill et al., 2017; Harvey et al., 2019). Alternatively, strong dispersal could simply impede differentiation and maintain genetically homogenous populations across wide species ranges. Using a comparative approach in reef fishes, Riginos, Buckley, Blomberg, and Treml (2014) showed that benthic guarders that disperse less than pelagic spawners tend to display greater degrees of population structure and species richness. This result establishes a continuity link between dispersal on the one hand, and diversification processes across both micro‐ and macro‐evolutionary timescales. At a broader scale, the mode of locomotion in vertebrate species was also shown to influence gene flow, with species that swim or fly tending to display weaker genetic structure than walking species (Medina, Cooke, & Ord, 2018). This finding, however, also raises the question of whether increased ability for long‐range dispersal could partly explain elevated species richness in flying vertebrates and fishes through increased colonization capacities of isolated habitats. As for the effective population size parameter, the extent and mechanisms by which the biological and ecological determinants of dispersal affect genetic connectivity remain a subject of ongoing research. By quantifying more precisely the evolutionary parameters related to effective migration and dispersal at different time scales, comparative genomics approaches have the potential to contribute significantly to these issues.

## 7 CONCLUSIONS

This review on the connectivity of spatially based evolutionary processes may be found surprising in its lack of consideration of the spatial dimension of the studied processes. This is by no means the sign of a lack of interest in the issue nor an attempt to devaluate spatially explicit approaches to identify environmental features and factors that affect connectedness across landscapes. This frustrating gap mainly reflects a limitation inherent to comparative studies, imposed by the need for a compromise between the extents of spatial and genome sampling resolution. This review deliberately took the path of an approach based on a limited number of genomes sampled in a few populations thought to be representative of the targeted processes. The main motivation for this choice comes from the wish to better connect micro‐ and macro‐evolutionary scales in connectivity research. However, recent developments in the analysis of genome‐wide genealogies combined with increasing sequencing capacities make it possible to foresee in the near future alternative approaches based on a random sampling of individuals through space (Bradburd & Ralph, 2019), toward a better integration of spatiality.

Connectivity disruptions caused by human activities cover a wide range of effects from habitat fragmentation to increased genetic exchanges between geographically isolated lineages within species. Depending on the context, the eco‐evolutionary aspects of connectedness that are the most relevant to conservation may impose different measures of connectivity. This may benefit from a reference‐based classification process, in an attempt to attend the conditions necessary for continuing evolution (Frankel & Soulé, 1981). For instance, the amount of connectivity that is required to limit demographic stochasticity is much higher than the one needed to avoid inbreeding depression, or insure genetic coupling for the maintenance of evolutionary potential. Ultimately, the comparative population genomics approach is expected to contribute to a quantitative assessment of the potential costs and benefits associated with facilitated or constrained dispersion. However, it is also important to remain clear about the real contributions of these approaches to combating biodiversity loss, bearing in mind that the most effective way to protect biodiversity is to stop threatening it wherever possible.

## CONFLICT OF INTEREST

None declared.

## Data Availability

Data sharing is not applicable to this article as no new data were created or analyzed in this study.
